# Supplementary material for: Towards unified AI-driven fracture mechanics: the extended deep energy method (XDEM)
Source: Nat Commun. 2026 Aug 15;17:8492. doi: 10.1038/s41467-026-76748-1 (PMC13484471; doi:10.1038/s41467-026-76748-1)
Supplement: Supplementary file 3 [file 41467_2026_76748_MOESM3_ESM.zip › pic/Results/Stress_intensity_factor/Cross/comparison_xdem_fem/angle_15/fem_dem_error_uv_3x2.pdf]

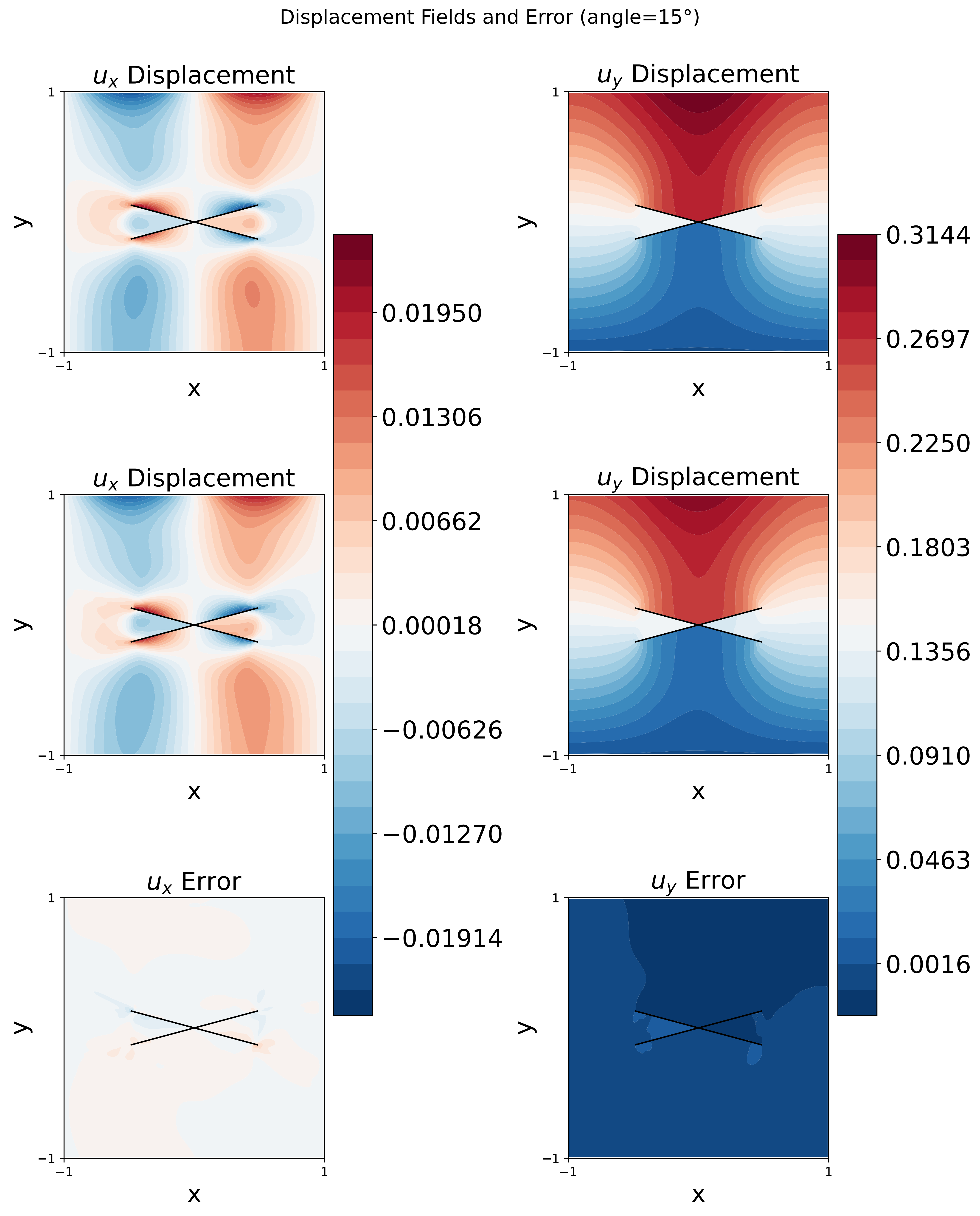

Displacement Fields and Error (angle=15°)
u_x Displacement
u_y Displacement
u_x Displacement
u_y Displacement
u_x Error
u_y Error
x
y
−1
1
0.01950
0.01306
0.00662
0.00018
−0.00626
−0.01270
−0.01914
0.3144
0.2697
0.2250
0.1803
0.1356
0.0910
0.0463
0.0016
